# BK Polyomavirus‐Associated Nephropathy Complicated by Life‐Threatening Hemorrhagic Pericardial Effusion Following Immunosuppression Reduction in a Kidney Transplant Recipient: A Case Report

**DOI:** 10.1155/crit/2999849

**Published:** 2026-07-29

**Authors:** Hazhir Moradi, Azar Naimi, Firouzeh Moeinzadeh

**Affiliations:** ^1^ Nosocomial Infection Research Center, Isfahan University of Medical Sciences, Isfahan, Iran, mui.ac.ir; ^2^ Reproductive Sciences and Sexual Health Research Center, Isfahan University of Medical Sciences, Isfahan, Iran, mui.ac.ir; ^3^ Isfahan Kidney Diseases Research Center, Isfahan University of Medical Sciences, Isfahan, Iran, mui.ac.ir

**Keywords:** case reports, graft survival, immunosuppressive agents, kidney transplantation, polyomavirus infections

## Abstract

**Background:**

BK polyomavirus (BKPyV) is an opportunistic infection following kidney transplantation due to immunosuppression. It is a recognized cause of tubulitis and graft dysfunction in kidney transplant recipients (KTRs). Management is challenging because clinicians must balance viral control with the risk of acute rejection. This report highlights the importance of individualized therapy in a 63‐year‐old KTR who developed BK polyomavirus‐associated nephropathy (BKPyVAN) after treatment for acute rejection.

**Case Report:**

A 63‐year‐old man with end‐stage renal disease secondary to long‐term NSAID use underwent a living‐donor kidney transplant at Alzahra Hospital, Isfahan, Iran. He was maintained on tacrolimus, mycophenolate mofetil (MMF), and prednisolone. At 5 months posttransplant, he developed acute cellular rejection treated with antithymocyte globulin (Thymoglobulin). Two months later, BKPyV viremia was detected (23,700 copies/mL). Immunosuppression was modified by switching tacrolimus to cyclosporine and discontinuing MMF. Two doses of intravenous immunoglobulin (IVIG; 2 g/kg total) were administered but discontinued due to fluid overload requiring temporary hemodialysis. Subsequently, the patient developed a large hemorrhagic pericardial effusion (7 × 12 cm) with left ventricular compression, likely attributable to a combination of fluid overload, uremia, and volume shifts during renal replacement therapy, requiring urgent pericardiocentesis with drainage of 550 mL of hemorrhagic fluid. By 10 months posttransplant, renal function stabilized (serum creatinine 2.3 mg/dL) and BKPyV PCR became undetectable.

**Conclusions:**

This case highlights a rare and life‐threatening complication, namely hemorrhagic pericardial effusion, arising during the management of BKPyVAN in a KTR. It underscores the importance of close cardiovascular monitoring, individualized immunosuppressive adjustment, and multidisciplinary care in this complex patient population.

## 1. Background

BK polyomavirus (BKPyV) is a nonenveloped, double‐stranded DNA virus of the Polyomaviridae family. Primary infection typically occurs during childhood and thereafter remains latent in the uroepithelial cells of immunocompetent individuals. In kidney transplant recipients (KTRs), reactivation of latent BKPyV under the pressure of immunosuppressive therapy may lead to BK polyomavirus‐associated nephropathy (BKPyVAN), characterized by tubulitis, interstitial inflammation, and progressive graft dysfunction [1–3]. BKPyV viruria is detected in up to 30% of KTRs, whereas viremia develops in approximately 10%; without intervention, up to 5%–8% progress to BKPyVAN [4].

The intensity of immunosuppression is the dominant risk factor for viral reactivation, particularly tacrolimus‐based regimens, antithymocyte globulin induction, and high‐dose corticosteroids [3, 5]. Recipient age, male sex, and BKPyV seronegativity at the time of transplantation may further increase susceptibility [5]. Host and donor BKPyV serostatus, as well as the degree of HLA mismatch, have also been implicated as contributing factors, though their relative importance remains an area of ongoing investigation.

At the pathological level, BKPyV replicates directly within renal tubular epithelial cells, causing cell lysis and progressive denudation of the tubular basement membrane. The resulting cytopathic changes, including nuclear enlargement, chromatin smudging, and intranuclear viral inclusion bodies, represent the histological hallmarks of active infection [6]. The inflammatory response triggered by this injury closely resembles acute cellular rejection both clinically and histologically, making accurate and timely diagnosis essential to guide appropriate management [7].

Reduction of immunosuppression remains the cornerstone of BKPyVAN treatment, yet this must be carefully individualized to avoid precipitating allograft rejection, a balance that is rarely straightforward in clinical practice [8, 9]. Several adjunctive agents, including intravenous immunoglobulin (IVIG), leflunomide, cidofovir, and fluoroquinolones, have been explored with variable success, and more recently, adoptive transfer of virus‐specific T cells (VSTs) has emerged as a promising therapeutic option in refractory cases [9, 10].

This report describes an unusual and clinically instructive case of BKPyVAN in a 63‐year‐old KTR who developed a life‐threatening hemorrhagic pericardial effusion during the course of immunosuppression reduction, a complication that, to our knowledge, has not been previously reported in this setting and that substantially shaped the management strategy.

## 2. Case Report

A 63‐year‐old male with a 20‐year history of ankylosing spondylitis, managed chronically with nonsteroidal anti‐inflammatory drugs (NSAIDs), developed end‐stage renal disease (ESRD) and underwent 2 years of hemodialysis. In January 2024, he received a living‐donor kidney transplant from an unrelated donor at Alzahra Hospital, Isfahan, Iran. Induction immunosuppression included antithymocyte globulin (Thymoglobulin, total dose 4 mg/kg over 3 days), methylprednisolone (6 mg/kg), and tacrolimus. Maintenance therapy consisted of tacrolimus, mycophenolate mofetil (MMF), and prednisolone.

During the first 5 months posttransplant, the patient maintained stable renal function with serum creatinine around 1.5 mg/dL. Monitoring was performed weekly during the first 2 months and biweekly thereafter. In accordance with institutional protocol, BKPyV viremia screening was performed monthly during the first 6 months posttransplant and every 3 months thereafter; all measurements during this initial period were negative. HLA typing revealed A2/A3, B35/B44, Cw4/Cw7, DRB101/11, and DQB103/06. Flow cytometric crossmatch was negative, and no donor‐specific antibodies (DSAs) were detected.

**Figure 1:**
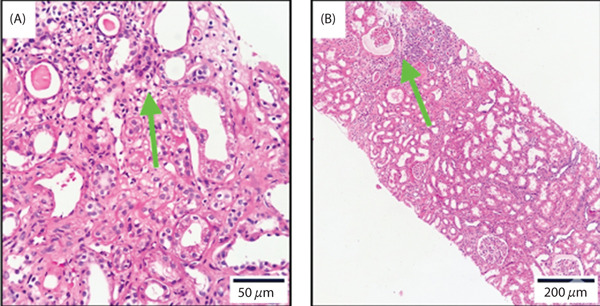
Histological findings from kidney allograft biopsy performed at 5 months posttransplant. (A) Moderate tubulitis (Banff t2) with lymphocytes infiltrating tubular epithelium. High‐magnification view (400 ×) highlighting areas of active tubulitis (green arrow). Scale bar = 50 *μ*m. (B) Overview of renal biopsy core showing interstitial inflammation (Banff i1) Low‐magnification overview (100 ×) demonstrating the extent of interstitial inflammatory infiltrates (green arrow). Scale bar = 200 *μ*m. (Hematoxylin and Eosin stain). Note: This figure illustrates histological evidence of acute cellular rejection (Banff Grade IA), without definitive nuclear changes of polyomavirus infection. No inclusion bodies were detected in this biopsy. Abbreviations: BKPyV – , BK polyomavirus; I – , interstitial inflammation; T, – tTubulitis.

In June 2024 (month five posttransplant), the patient presented with a sudden rise in serum creatinine to 2.2 mg/dL. Tacrolimus trough level was 8 ng/mL. BKPyV viremia tested at this time point was negative by PCR. A kidney allograft biopsy confirmed acute cellular rejection (Banff IA) on hematoxylin and eosin (H&E) staining; no cytopathic changes suggestive of viral infection were identified (Figure 1). He received Thymoglobulin (4 mg/kg) for treatment and was discharged with improved renal function (creatinine 1.8 mg/dL).

In August 2024 (month seven posttransplant), serum creatinine rose again to 2.4 mg/dL. Testing showed negative cytomegalovirus (CMV) PCR but positive BKPyV PCR with a viral load of 23,700 copies/mL. Immunosuppressive therapy was modified by switching tacrolimus to cyclosporine and discontinuing MMF. Two doses of IVIG (1 g/kg each) were administered.

However, the patient developed fluid overload requiring temporary hemodialysis. A transthoracic echocardiogram revealed a 7 × 12 cm hemorrhagic pericardial effusion compressing the left ventricle. Pericardiocentesis was performed, yielding 550 mL of hemorrhagic fluid, followed by 5 days of continuous drainage. No infection was identified in the pericardial fluid.

The effusion was attributed to a combination of fluid overload from IVIG administration, volume shifts during renal replacement therapy, and uremia, rather than an infectious or malignant etiology. The patient had no prior history of pericarditis or cardiovascular disease. Repeat kidney allograft biopsy was deferred given the patient′s clinical instability at that time.

By October 2024 (month 10 posttransplant), the patient′s urine output improved and dialysis was discontinued. His serum creatinine stabilized around 2.3 mg/dL, and BKPyV PCR was negative. He continues on cyclosporine (target trough level approximately 110 ng/mL) with monthly clinic follow‐up. BKPyV viremia is monitored every 3 months, and graft function has remained stable to date.

## 3. Discussion

This case illustrates the multifaceted challenges encountered when managing BKPyVAN in a KTR who had previously received intensified immunosuppression for acute cellular rejection. Beyond the well‐recognized difficulty of balancing viral control with rejection prevention, our patient experienced a life‐threatening hemorrhagic pericardial effusion during the course of treatment, a complication that has rarely been described in this context and that required urgent intervention. This case therefore offers insights that extend beyond standard BKPyVAN management.

BKPyV reactivation is a well‐recognized complication in the posttransplant setting, with up to 10% of patients developing viremia and approximately 5% progressing to BKPyVAN [1, 2]. Risk factors include the degree and duration of immunosuppression, especially tacrolimus‐based regimens, as well as recipient factors such as older age, male sex, and the degree of immunosuppressive exposure [3]. In our patient, advanced age, male sex, and intensified immunosuppression with Thymoglobulin after acute rejection were important contributing risk factors for viral reactivation; pretransplant BKPyV serostatus was unavailable for either the recipient or the donor, which is not uncommon in resource‐limited settings and may limit risk stratification prior to transplantation.

Histologic diagnosis of BKPyVAN is based on characteristic cytopathic changes in renal tubular epithelial cells, including viral inclusion bodies and nuclear enlargement [4]. However, in our case, the definitive diagnosis was made clinically based on viremia and graft dysfunction, as the biopsy did not show classic viropathic changes. SV40 large T antigen immunohistochemistry, which represents the gold standard for histological confirmation of BKPyVAN, was not available at our institution at the time of biopsy. This is not uncommon and has been reported in previous studies, particularly during early stages of viral reactivation [5]. Similar observations have been described in other case reports where viremia was detected without histological inclusion bodies, especially in early BKPyVAN. These reports emphasize the importance of integrating clinical and virological findings when histological confirmation is inconclusive or unavailable.

The cornerstone of BKPyVAN treatment remains reduction of immunosuppression [6]. In our case, tacrolimus was replaced with cyclosporine, and MMF was withdrawn. This strategy is supported by recent observational data and expert guidelines [7, 8]. Previous reports have described variable outcomes with IVIG use in BKPyVAN, with some cases showing reduced viral load whereas others reported no significant benefit. In our patient, IVIG was discontinued due to fluid overload, highlighting the potential risks of this adjunctive therapy. Other adjunctive agents reported in the management of BKPyVAN include fluoroquinolones, leflunomide, and cidofovir, each with limited and largely anecdotal evidence supporting their use [2]. More recently, adoptive transfer of VSTs has been described as a promising option in severe or refractory cases, offering a targeted approach to restore antiviral immunity without broadly increasing immunosuppression [10].

A particularly striking feature of this case was the development of a large hemorrhagic pericardial effusion requiring urgent pericardiocentesis. To our knowledge, this complication has not been previously reported in the context of BKPyVAN management, rendering this case clinically unique. The etiology was likely multifactorial. Infectious causes were excluded by pericardial fluid analysis. Malignancy was not suspected given the clinical context. The most probable contributing factors were fluid overload following IVIG administration, volume shifts during renal replacement therapy, and uremia‐associated pericarditis in the setting of acute kidney injury. The absence of prior cardiovascular disease or pericarditis in this patient further supports an acquired, treatment‐related pathogenesis. This complication underscores the critical importance of cardiovascular monitoring and early nephrology‐cardiology collaboration when managing BKPyVAN in patients with concurrent renal insufficiency.

The challenges encountered in this case are consistent with those described in the broader BKPyVAN literature. Shen et al. [6] highlighted the difficulty of simultaneously controlling viral replication and preventing rejection, whereas the second international consensus guidelines on BKPyV management emphasize individualized immunosuppressive adjustment based on virological and clinical response [3]. Our patient′s outcome aligns with these recommendations.

Ultimately, this case extends beyond the typical BKPyVAN narrative by documenting a rare and life‐threatening cardiovascular complication arising during immunosuppression reduction. It reinforces the value of routine BKPyV screening, early therapeutic adjustment, and close multidisciplinary monitoring, particularly in patients at heightened risk of fluid overload or uremic complications.

## 4. Conclusions

Early identification of BKPyV reactivation and prompt modification of immunosuppression remain essential to limit viral replication while minimizing the risk of acute rejection. This case further demonstrates that the management of BKPyVAN may be complicated by rare but life‐threatening events, such as hemorrhagic pericardial effusion, particularly in patients receiving adjunctive therapies in the setting of renal insufficiency. Even in the absence of definitive histologic confirmation, integrating clinical and virological findings with timely therapeutic adjustments can preserve graft function. Close cardiovascular monitoring and early multidisciplinary involvement are strongly recommended in this patient population.

**Nomenclature**
AKI: acute kidney injury
BKPyV: BK polyomavirus
BKPyVAN: BK polyomavirus‐associated nephropathy
CMV: cytomegalovirus
DSA: donor‐specific antibodies
ESRD: end‐stage renal disease
HLA: human leukocyte antigen
IVIG: intravenous immunoglobulin
KTR: kidney transplant recipient
MMF: mycophenolate mofetil
NSAID: nonsteroidal anti‐inflammatory drug
VST: virus‐specific T cells

## Author Contributions

H.M. drafted the manuscript; A.N. contributed to literature review; F.M. supervised patient management and critically revised the manuscript.

## Funding

No funding was received for this manuscript.

## Disclosure

All authors have read and approved the final version of the manuscript.

## Ethics Statement

Written informed consent to participate was obtained from the patient. No donor family or legally authorized representative consent was required, as the living donor provided consent directly prior to transplantation. No organs from executed prisoners or other vulnerable populations were used.

## Consent

Written informed consent was obtained from the patient for publication of this report.

## Conflicts of Interest

The authors declare no conflicts of interest.

## Data Availability

Data sharing not applicable to this article as no datasets were generated or analyzed during the current study.
